# *SEPTIN12* Genetic Variants Confer Susceptibility to Teratozoospermia

**DOI:** 10.1371/journal.pone.0034011

**Published:** 2012-03-30

**Authors:** Ying-Hung Lin, Ya-Yun Wang, Hau-Inh Chen, Yung-Che Kuo, Yu-Wei Chiou, Hsi-Hui Lin, Ching-Ming Wu, Chao-Chin Hsu, Han-Sun Chiang, Pao-Lin Kuo

**Affiliations:** 1 Graduate Institute of Basic Medicine, Fu Jen Catholic University, College of Medicine, Taipei, Taiwan; 2 Department of Obstetrics & Gynecology, National Cheng Kung University, College of Medicine, Tainan, Taiwan; 3 Institute of Molecular Medicine, National Cheng Kung University, College of Medicine, Tainan, Taiwan; 4 Department of Biochemistry and Molecular Biology, National Cheng Kung University, College of Medicine, Tainan, Taiwan; 5 Graduate Institute of Basic Medical Sciences, National Cheng Kung University, College of Medicine, Tainan, Taiwan; 6 Department of Biomedical Engineering, National Cheng Kung University, College of Engineering, Tainan, Taiwan; 7 Department of Physiology, National Cheng Kung University, College of Medicine, Tainan, Taiwan; 8 Department of Cell Biology & Anatomy, National Cheng Kung University, College of Medicine, Tainan, Taiwan; 9 Department of Obstetrics and Gynecology, China Medical University, Taichung, Taiwan; Baylor College of Medicine, United States of America

## Abstract

It is estimated that 10–15% of couples are infertile and male factors account for about half of these cases. With the advent of intracytoplasmic sperm injection (ICSI), many infertile men have been able to father offspring. However, teratozoospermia still remains a big challenge to tackle. Septins belong to a family of cytoskeletal proteins with GTPase activity and are involved in various biological processes e.g. morphogenesis, compartmentalization, apoptosis and cytokinesis. *SEPTIN12*, identified by c-DNA microarray analysis of infertile men, is exclusively expressed in the post meiotic male germ cells. *Septin12^+/+^/Septin12^+/−^* chimeric mice have multiple reproductive defects including the presence of immature sperm in the semen, and sperm with bent neck (defect of the annulus) and nuclear DNA damage. These facts make *SEPTIN12* a potential sterile gene in humans. In this study, we sequenced the entire coding region of *SEPTIN12* in infertile men (n = 160) and fertile controls (n = 200) and identified ten variants. Among them is the c.474 G>A variant within exon 5 that encodes part of the GTP binding domain. The variant creates a novel splice donor site that causes skipping of a portion of exon 5, resulting in a truncated protein lacking the C-terminal half of SEPTIN12. Most individuals homozygous for the c.474 A allele had teratozoospermia (abnormal sperm <14%) and their sperm showed bent tail and de-condensed nucleus with significant DNA damage. *Ex vivo* experiment showed truncated SEPT12 inhibits filament formation in a dose-dependent manner. This study provides the first causal link between *SEPTIN12* genetic variant and male infertility with distinctive sperm pathology. Our finding also suggests vital roles of SEPT12 in sperm nuclear integrity and tail development.

## Introduction

### Male infertility

Between 10% and 15% of couples worldwide are affected by reduced fertility, and the defects can be traced to the men in roughly half of the cases [1]. The pathology of male infertility includes anatomic defects, gametogenesis dysfunction, endocrinopathies, immunologic problems, ejaculatory failure, environmental exposures, and gene mutations [2], [3]. During the past two decades, the development of intracytoplasmic sperm injection (ICSI) has changed the treatment of male infertility [4]. Although the ICSI technique is a breakthrough for assisted reproduction, many infertile couples are still unable to achieve paternity through testicular sperm extraction (TESE) and ICSI [5]. Recently, several studies indicated sperm DNA damage is associated with ICSI failure, development arrest of preimplantation embryos and high rates of miscarriage [3], [4], [5].

### Septins

Septins belong to a highly conserved family of polymerizing GTP binding proteins [6], [7]. They were initially identified in the budding yeast, *Saccharomyces cerevisia*. Loss of function for any one of the five septins, Cdc3p, Cdc10p, Cdc11p, Cdc12p, and Shs1p/Sep7p, which are localized to the ring(s) of mother and budding daughter cells, results in multi-nuclear morphology [8], [9]. There are 14 septin genes in mammalian species, and most of them generate multiple splice isoforms [6], [10]. Some septins are expressed ubiquitously, while others are only expressed in well-differentiated cells (e.g. neuron or male germ cells) [6]. In well-differentiated cells, septins are involved in vesicle trafficking and boundary formation [11], [12], [13]. For example, SEPT3 and SEPT5 are solely expressed in neurons, localized in presynaptic terminal and with synaptic vesicles [14], [15]. However, *Septin 3^−^* ^/−^ and *Septin 5^−/−^* mice do not show any overt neurological phenotypes [16], [17]. Besides, growing evidence has suggested mammalian septins interact with diverse molecules to ensure completion of cytokinesis in somatic cells, but the underlying mechanisms still remain elusive [18], [19]. In somatic cells, SEPT2, SEPT6, SEPT7 and SEPT9 have been implicated in the completion of cytokinesis in dividing cells [18], [19], [20], [21]. SEPT7 also interacts with centromere protein E (CENP-E) for stable CENP-E localization to the kinetochore and for achieving chromosome alignment at the equator during cytokinesis [19]. In cell models, knockdown of SEPTIN2, SEPTIN6, SEPTIN7 or SEPTIN9 causes high percentage of cells with two nuclei [18], [22], [23]. However, *Septin 6*-deficient mice were grossly normal and did not exhibit abnormal phenotypes [24]. Roles of SEPT2, 7 and 9 in the animals are still not demonstrated.

### The role of SEPTs in male reproduction

In *Drosophila*, SEPTs (*Pnut*, *Sep1*, and *Sep2*) are involved in the formation of ring canal structure between the intercellular bridge of male and female germ cells [25]. In the mammalian species, SEPT2, 7 and 9 have been found to co-localize with an intercellular bridge marker of male germ cells, TEX14 (*testis-expressed gene14*) [26]. Loss of TEX14 in mice cause disruption of intercellular bridge as well as increased apoptosis of germ cells [27]. SEPT4, along with other SEPTs (SEPT1, SETP6, and SEPT7), is located at the annulus, a ring-like structure between the midpiece and the tail region of spermatozoa [12], [13]. *Septin 4* null mice were viable but sterile in males; the immotile sperm had defective annulus and showed dis-localization of SEPT1, SEPT6 and SEPT7 from the annulus [12], [13]. Disorganized annulus/septin rings were also found in a subset of human patients with asthenozoospermia [12], [28], [29].

### SEPTIN12

We have used microarray analysis to search for genes that are potentially involved in human spermatogenic defects. Of ten novel testis-specific genes thus identified, one was *SEPTIN12* (MIM* 611562) [30]. In rat, SEPT12 is found at the annulus of mature spermatozoa [31]. We also found that in humans SEPT12 is expressed in different subcellular compartments of post-meiotic germ cells, including the head and neck of spermatids and the annulus of mature sperm [32]. By knocking out one allele of *Septin12* in the mouse, we found that haploinsufficiency of *Septin12* results in male sterility [32]. Sperm obtained from *Septin12* ^+/+^/*Septin12^+/−^* chimeric mice reveals multi-defects including immature sperm, bent- neck with disrupted annulus, and nuclear DNA damage [32], [33]. Considering SEPT12 is exclusively expressed in the testis and its expression level is critical for male germ cell development, *SEPTIN12* has emerged as an interesting candidate for male sterile gene.

### Filament formation of SEPTs via polymerization

SEPTs usually mediate their cellular function through the formation of macromolecular and hetero-oligomeric filaments both *in vivo* and *in vitro* [21], [34]. Biochemical methods have been used to isolate at least three SEPT complexes: SEPT2/6/7 [34], SEPT7/9b/11 [35] and SEPT4/5/8 [36]. The filament-like structure has been observed in many SEPTs [18], [20], [22] and loss of a SEPT subunit may affect the stability of other subunits in the same complex [18], [34], [37]. We previously found that SEPT12 forms long filaments both *in vitro* (293T cells) and *in vivo* (round spermatids) [32], [33]. SEPT12 also interacts with SEPT6 and SEPT11 and forms filaments in Hela cells [38], [39]. In the mouse, SEPT1/4/6/7 may be assembled to form a circular-like structure at the annulus of mature sperm [13].

In this study, we sequenced the entire coding sequences of *SEPTIN12* in infertile men with abnormal semen parameters and identified ten SNPs. One of them, c.474 G>A, is more prevalent in infertile men than control subjects. This SNP, c.474G>A, located at exon 5 within the GTP binding domain, may create a novel alternative splicing variant by the activation of a cryptic splice donor. The novel transcript leads to the translation of a truncated protein that lacks partial exon 5 and exons 6–10. The truncated SEPT12 may disturb the filament formation of wild-type SEPT12. Infertile men carrying this SNP are presented with distinctive sperm pathology, including de-condensed nucleus with significant DNA damage, bent tail or loss of tail. Our findings provide the first clue about a causal link between *SEPTIN12* genetic variant and male infertility with distinctive sperm pathology.

## Results

### Identification of genetic variants in SEPTIN12

**Figure 1:**
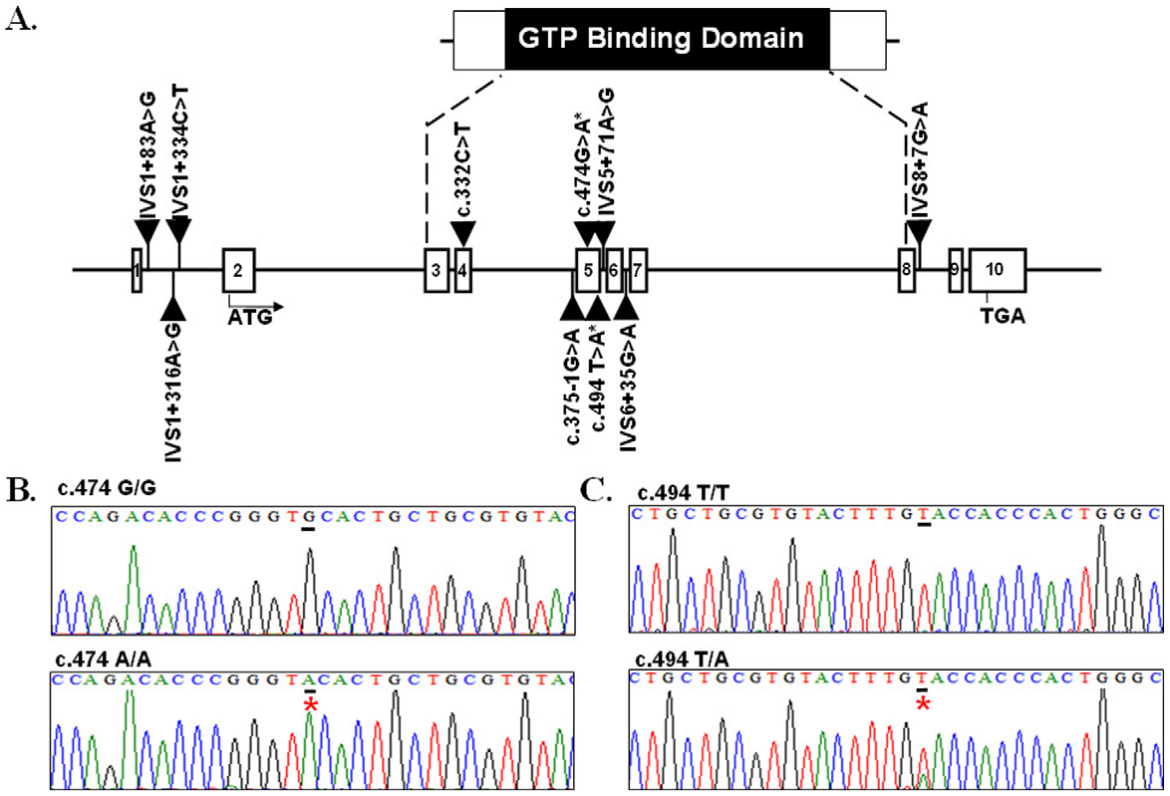
Identification of novel variants in the SEPTIN12 gene. Genomic structure of the *SEPTIN12* gene and positions of the ten SNPs. Open bars indicate exons. The ATG start site is located at exon 2. Exon 3 to exon 8 encodes the GTP -Binding Domain of SETIN12. (B.–C.) Electropherograms showing DNA sequences. Lower panels show the variant (c.474C→A, Left; c.494T→A, Right) sequences, whereas the upper panels show the wild-type (normal) sequences. Red stars indicate locations of the variants.

A total of 160 infertile men with abnormal semen parameters and 200 fertile controls were subjected to *SEPTIN12* sequence analysis. Ten SNPs identified included seven intronic variants (IVS1+83A>G, IVS1+316A>G, IVS1+334C>T, c.375−1G>A, IVS5+71A>G, IVS6+35G>A and IVS8+7G>A), one synonymous variant (c.474G>A) and two non-synonymous variant (c.332C>A, *pThr111Lys*; c.494T>A, *p.Val165Gln*). Their locations are shown in Figure 1A. Six SNPs are located between exon 3 to exon 8, which encode the GTP binding domain critical for the polymerization of SEPT. They are c.332C>A, c.375−1G>A, c.474G>A, c.494T>A, IVS5+71A>G and IVS6+35G>A (Figure 1A). Both allele and genotype frequencies of c.474 A were more prevalent in the infertile men (*p*  = 0.007 and 0.003, respectively) (Figure 1B and Table 1). Another SNP, c.494T>A SNP, was more prevalent in the control subjects (*p* = 0.032 and 0.013, respectively) (Figure 1B and Table 1).

**Table 1:** SEPTIN12 allele frequencies in infertile men and control subjects.

SNP	Allele frequency				Genotype frequency (%)			
	Allele	Control (n = 400) (%))	Spermatogenic failure (n = 320) (%)	*p*	Genotype	Control (n = 200) (%)	Spermatogenic failure (n = 160) (%)	*p*
IVS1+ 83A>G	A	279 (69.8%)	219 (68.4%)	0.705	AA	93(46.5%)	80 (50%)	0.060
	G	121(30.2%)	101(31.6%)		AG	93(46.5%)	59 (37% )	
					GG	14 (7%)	21 (13%)	
IVS1+ 316A>G	A	307 (76.8%)	239 (74.7%)	0.521	AA	117 (58.5%)	91 (56.9%)	0.616
	G	93 (23.2%)	81 (25.3%)		GA	73(36.5%)	57 (35.6%)	
					GG	10 (5%)	12(7.5%)	
IVS1+ 334C>T	C	395 (98.7%)	315(98.4%)	0.722	CC	195 (97.5%)	155 (96.9%)	0.720
	T	5 (1.3%)	5( 1.6%)		CT	5(2.5%)	5 (3.1%)	
					TT	0 (0%)	0 (0%)	
c.332 C>A	C	394(98.5%)	318 (99.4%)	0.266	CC	194 (97%)	158 (98.7%)	0.263
	A	6(1.5%)	2 (0.6%)		CA	6 (3%)	2 (0.3% )	
					AA	0(0%)	0 (0%)	
c.375− 1G>A	G	383 (95.7%)	297 (92.8%)	0.087	GG	183 (91.5%)	137 (85.6%)	0.078
	A	17 (4.3%)	23 (7.2%)		GA	17 (8.5%)	23 (14.4%)	
					AA	0 (0%)	0 (0%)	
c.474 G>A	G	360(90%)	266 (83.1%)	0.007**	GG	163 (81.5%)	121(75.6%)	0.003**
	A	40(10%)	54( 16.9%)		GA	34(17%)	24(15.0%)	
					AA	3(1.5%)	15(9.4%)	
c.494 T>A	T	304 (76%)	264 (96%)	0.034*	TT	104(52%)	104 (65%)	0.013*
	A	96(24%)	56(4%)		TA	96(48%)	56 (35% )	
					AA	0(0%)	0 (0%)	
IVS5+ 71A>G	A	386 (96.5%)	311 (97.2%)	0.602	AA	187 (93.5%)	153 (95.6%)	0.333
	G	14 (3.5%)	9( 2.8%)		AG	12 (6%)	5 (3.1%)	
					GG	1(0.5%)	2 (1.3%)	
IVS6+ 35G>A	G	385 (96.2%)	302 (94.4%)	0.232	GG	185 (92.5%)	146 (91.2%)	0.074
	A	15 (3.8%)	18 (5.6%)		GA	15 (7.5%)	10 (6.3%)	
					AA	0 (0.0%)	4 (2.5%)	
IVS8+ 7G>A	G	392(98.0%)	312 (97.5%)	0.651	GG	192(96.0%)	152 (95.0%)	0.647
	A	8 (2.0%)	8 (2.5%)		GA	8(4.0%)	8 (5.0%)	
					AA	0(0.0%)	0(0.0%)	

Nucleotide numbering indicates cDNA numbering with 1+ corresponding to the A of the ATG translation initiation codon in the reference cDNA sequence of *SEPTIN12* (**NM_144605.3**).

### Spermatozoa from patients who carried c.474 A/A showed distinct morphological defects

**Figure 2:**
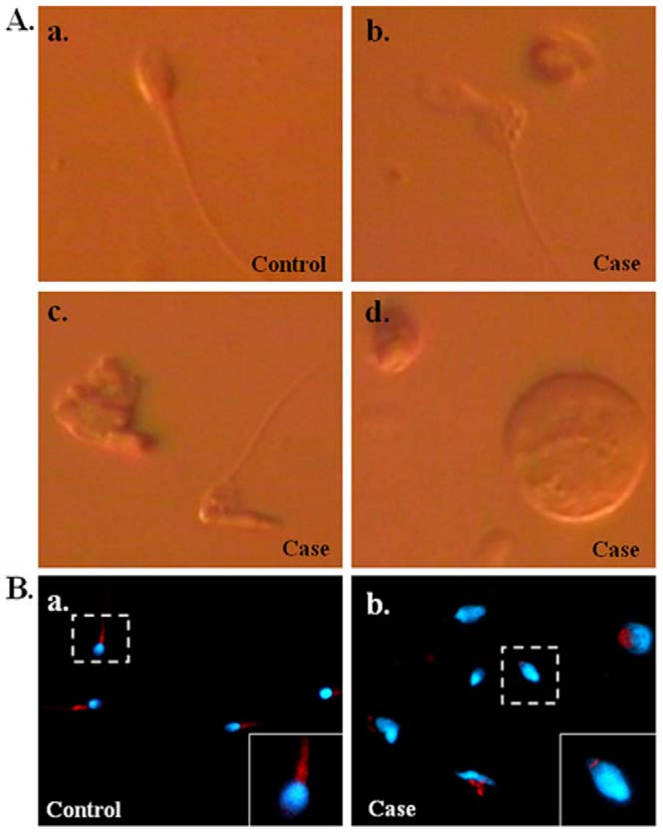
Abnormal morphology of spermatozoa from a case with c.474A/A. (A.) Motile sperm organelle morphology examination (MSOME) for sperm cells using a high-magnification inverted microscope (magnification was ×8400). (a.) sperm from a fertile control with c.474C/C; (b.–d.) sperm from an infertile man with c.474A/A. Sperm with bent-tail (b.), neck(c.) and round head (d.). (B.) IFA assay for sperm from a fertile control with c.474C/C (a.) and an infertile man with c.474A/A (b.). DAPI: blue; mito-tracker: red. (Magnification: ×400).

In this study, 9 of the 15 infertile men with c.474A/A were presented with teratozoospermia (88%–99% of abnormal sperm) (Table 2). To detail the morphological pattern of their spermatozoa, motile sperm organelle morphology examination (MSOME) and immuno-fluorescence assay (IFA) were performed. Most sperm were found to have distinct pathological features, including bent-tail, head with abnormal shape and immature spermatid (Figure 2A and 2B).

**Table 2:** Clinical features of the 15 men with the c.474 A/A genotype.

Patient NO.	Age (years)	Sperm count (×10^6^/ml)	Abnormal Sperm (%)	c.474 G/A
1	39	186.0	91	AA
2	33	28.4	88	AA
3	35	20.4	99	AA
4	38	66.0	92	AA
5	38	24.0	94	AA
6	38	91.0	92	AA
7	35	27.7	97	AA
8	43	109.0	93	AA
9	37	1.3	97	AA
10	37	Azoospermia	–	AA
11	31	Azoospermia	–	AA
12	30	Azoospermia	–	AA
13	34	Azoospermia	–	AA
14	34	Azoospermia	–	AA
15	25	NA	NA	AA

### The c.474 G to A transition may activate a cryptic splice donor site

**Figure 3:**
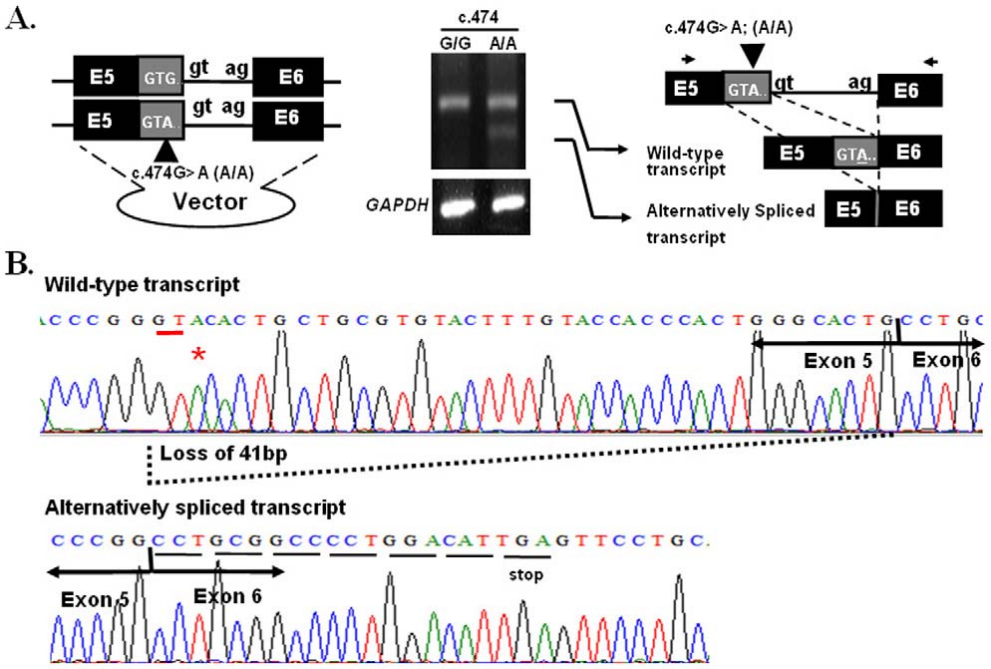
Effects of the c.474G→A variant on the splicing process. (A.) The c.474 G→A SNP induces alternative splicing *ex vivo*. Left panel: PCR fragments containing exon 5 (E5, back and gray box), intron 5 (within splice donor site “gt” and splice acceptor site “ag”) and exon 6 (E6, black box) with c.474 G or c.474A were constructed into a vector, respectively. Middle panel: products of RT–PCR are shown on an agarose gel. Control: *GAPDH* Two transcripts (wild-type and alternatively spliced)are produced by the minigene with c.474A. Right panel: schematic depiction of the RT–PCR products. Wild-type transcript: using the original splice donor and acceptor site. Alternatively spliced transcript: using the novel splice donor induced by c.474G→A and the original splice acceptor site. (B.) Sequences of the wild-type (upper panel) and alternatively spliced (lower panel) products from the minigene containing c.474A. The alternatively spliced transcript induces a novel splice donor site, which results in skipping of partial exon 5 (with loss of 41 bp), and also created a premature stop codon in exon 6.

To evaluate the functional effect of c.474G→A, *ex vivo* assay was preformed in the NTERA-2 d.D1 (NT2D1) cell line, a pluripotent human testicular embryonal carcinoma cells. The cell line was transfected with plasmids containing partial *SEPTIN12* (exon 5, intron 5, exon 6, intron 6 and exon 7) with either c.474G/G or A/A. RT-PCR analysis of cells transfected with the c.474 A/A plasmid showed a smaller minor transcript that was absent in cells transfected with the c.474 G/G plasmid (Figure 3A). The ratio between the wild-type and alternatively spliced transcript was about 1.7 to 1. Sequencing analysis showed that the G to A transition created a novel splice donor site and resulted in the skipping of the 3′ portion of exon 5 (loss of 41 bp) and the shift to a new reading frame with a premature stop codon in exon 6 (Figure 3B). The novel transcript therefore encodes a truncated protein lacking the C-terminal half of SEPT12, including part of the GTP binding domain.

### Truncated SPET12 disrupts polymerization of wild-type SEPT12 and nuclear membrane localization

**Figure 4:**
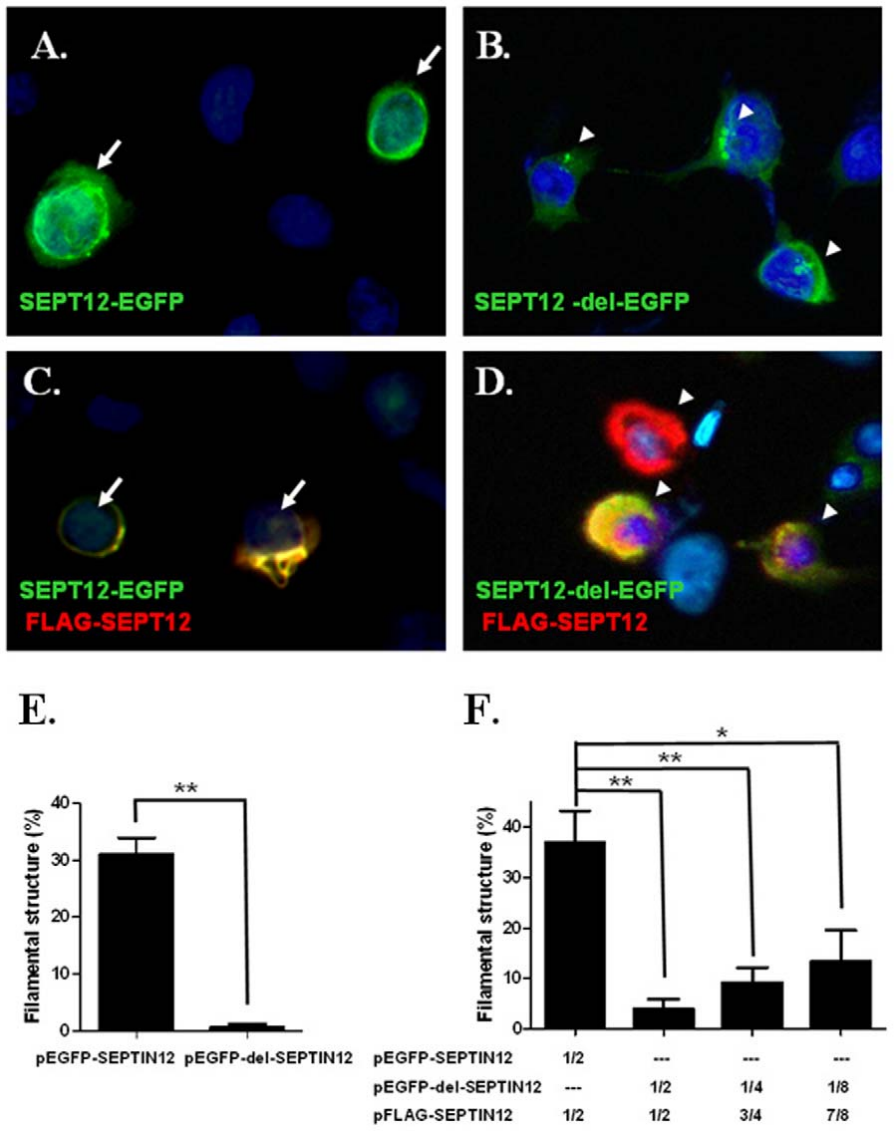
Effects of truncated - SEPT12 on filament - like formation in NT2D1 cells. Immuno-fluorescence assay (IFA) shows wild- SEPT12-EGFP (SEPT12-EGFP) (A.) or truncated- SEPT12-EGFP (SEPT12-del-EGFP) (B.) forms filament - like or dot- like structure, respectively. (A.–B.) Merged pictures for staining with anti-EGFP antibody (green) and DAPI (light blue). The results of co-expressed wild- SEPT12-EGFP (SEPT12-EGFP) (C.) or truncated- SEPT12-EGFP (SEPT12-del-EGFP) (D.) with wild- FLAG-SEPT12 (FLAG-SEPT12) in cells are presented in (C.) and (D.), respectively. Signals from EGFP protein (green), anti-FLAG antibody (red) and DAPI (Light blue) signals are merged in (C.) and (D.). (A.–D.) Arrows indicated filament-like structure; Arrow head indicated dot-like structure. Magnification: ×400 in A–D. (E.–F) Quantification of the percentage of filament- like structures in transfected cells. The height of the boxes represents the mean of value obtained from four independent experiments. At least 100 transfected cells were counted in each experiment (**: *p*<0.01, Student's t test). (F.) Dosage- dependent inhibition of filament-like formation by the truncated SEPT12 protein. Plasmids encoding FLAG-tagged wild-type SEPTIN12 were mixed with various amounts of plasmids encoding EGFP-tagged wild- or truncated SEPTIN12, then the mixtures were transfected into NT2D1 cells (**: *p*<0.01,*: *p*<0.05, Student's t test).

To determine the functional significance of the truncated SEPT12 protein (SEPT12-del-EGFP) generated from the c.474A/A allele, plasmids encoding SEPT12-EGFP and SEPT12-del-EGFP were transfected into NT2D1 cells, respectively. Over-expressed SEPT12-EGFP formed filaments surrounding the nuclear membrane (Figure 4A.). However, over-expressed SEPT12-del-EGFP aggregated to form a dot- like structure that did not surround the nuclear periphery (Figure 4B and 4E). To test the hypothesis that the mutant protein may influence the function of wild-type protein, we co-transfected cells with SEPT12-del-EGFP and FLAG-SEPT12 expression vectors in different ratios, 1∶ 1, 1∶ 3 and 1∶7. Cells co-transfected with FLAG-SEPT12 and SEPT12-EGFP formed well polymerized filaments around the nuclear membrane (Figure 4C). However, SEPT12-del-EGFP disrupted the filament formation of FLAG-SEPT12 in a dose-dependent manner (Figure 4D and 4F). The finding suggests that truncated SEPT12 lacking the C-terminal may disrupt the polymerization (and filament formation) of the wild-type SEPT12.

### Disruption of SETP12 filament in spermatozoa of infertile men with c.474 A/A

**Figure 5:**
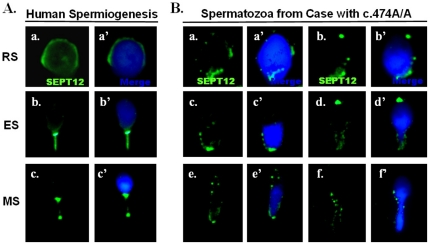
Expression patterns of SEPT12 human male germ cells with the c.474G/G (wild) and c.474A/A genotypes. (A.) Detection of SEPT12 signals during human spermiogenesis. (a.–a′): Round Spermatids (RS), (b.–b′) Elongating Spermatids (ES) and Mature Sperm (MS). Left: SEPT12 signal (green); Right: merge of SEPT12 (green) and DAPI (light blue) signals. (B.) Varied type of spermatozoa isolated from cases with the c.474A/A genotype. Left: SEPT12 signal (green); Right: merge of SEPT12 (green) and DAPI (light blue) signals (Magnification: ×1,000).

To determine whether del-SEPT12 also affects terminal differentiation of male germ cells in humans, spermatozoa were isolated from the testis biopsies of fertile controls with c.474C/C and infertile men with c.474A/A. IFA showed that in fertile men, SEPT12 is present around the nuclear periphery of round spermatids, at the neck region of elongating spermatids, and at the neck region and annulus of mature spermatozoa (Figure 5A), a finding in accord with our previously report [33]. In germ cells isolated from infertile men who carried c.477A/A, SEPT12 showed a dot-like pattern in differential stages of haploid germ cells (Figure 5B). This finding is in accord with the expression pattern of SEPT12-del-EGFP in NT2D1 cells (Figure 4B and 4D).

### De-condensed sperm nucleus and increased DNA damage in infertile men with c.474 A/A

**Figure 6:**
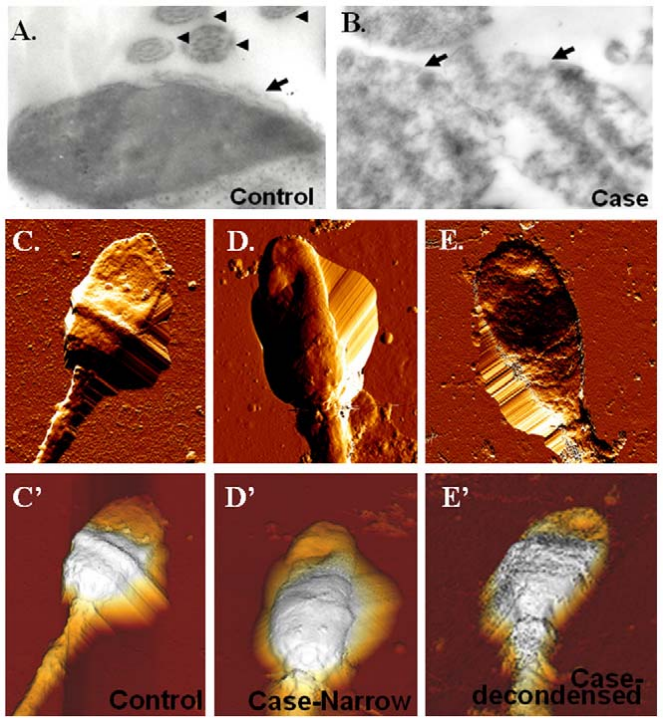
Spermatozoa from c.474A/A patients with abnormal head shape. (A.–B.) TEM images of sperm isolated from a fertile control (A.) and an infertile mam with c.474A/A (B). The latter shows de-condensed chromatin. Arrows indicate the nucleus; arrow heads indicate the axonemal 9+2 structures (Magnification: ×10,000). (C.–E.) Top-view AFM images confirm abnormal morphology in sperm head. Sperm of a control subject (C.). Sperm of an infertile man with c.474A/A have a narrow head (D.) or a de-condensed nucleus (E.). Three-dimensional images are displayed in the bottom (C′.–E′.).

**Figure 7:**
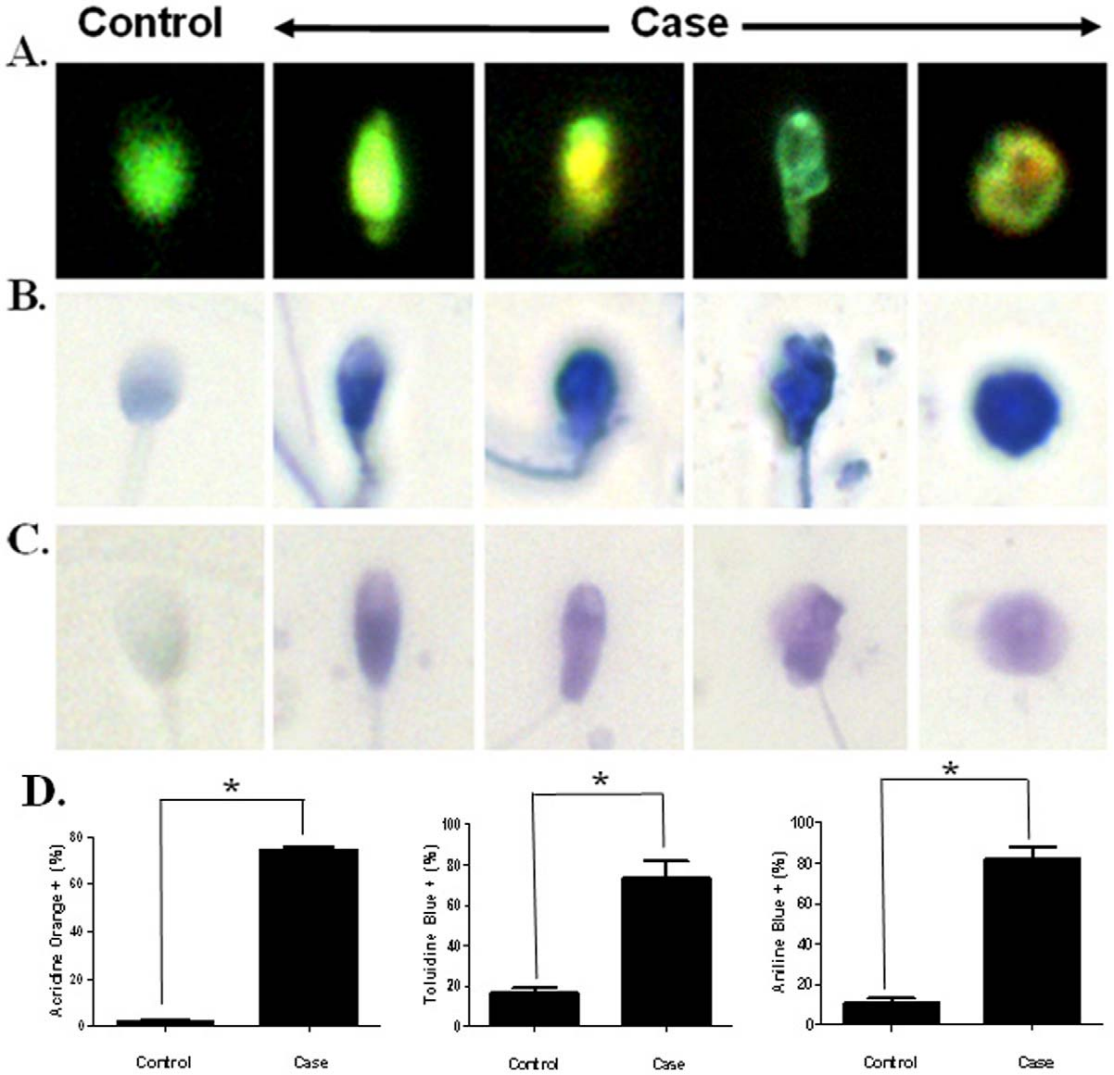
Nuclear DNA damage in the spermatozoa of infertile men who carried c.474A/A. (A.–C.) The spermatozoa were stained with AO (A.), TB (B.) and AB (C.) dyes. (A.) The spermatozoa with normal (green) or abnormal nucleus (yellow). (B.–C.) Spermatozoa with normal (light blue) or abnormal (dark blue) nucleus. (Magnification: ×1,000). (D.) Quantification of the percentage of AO-, AB- or TB- stained spermatozoa. At least 100 spermatozoa cells were counted in each case (*: *p*<0.05; Mann-Whitney test).

Increased sperm nuclear DNA damage has been observed in abnormal sperm of *Septin12^+/+^/Septin12^+/−^* chimeric mice [33]. In addition, oocytes couldn't develop beyond the morula stages after IVF or ICSI using sperm obtained from the *Septin12^+/+^/Septin12^+/−^* chimeric mice [33]. To evaluate sperm nuclear integrity of infertile men with c.474A/A, transmission electron microscopy (TEM) and atomic force microscopy (AFM) were used. Sperm from c474A/A patients had loose nuclear matrix as examined by TEM (Figure 6A and 6B) and narrow head/de-condensed nuclear matrix as observed under AFM (Figure 6 D and E). These two classical phenotypes have been described in a previous study using AFM to examine human sperm [40]. Further, we found high percentage of sperm with nuclear DNA damage by AO, TB and AB staining (AO: P<0.05; TB: P<0.05; AB: p<0.05; by Mann-Whitney test) (Figure 7 A–D). Taken together, we found SEPT12 dysfunction caused by c.474A/A may disrupt the nuclear integrity, a finding reminiscent of that observed in the *Septin12^+/+^/Septin12^+/−^* chimeric mice.

## Discussions

In this study, a *SEPTIN12* genetic variant (c.474G→A) was found to be significantly associated with male infertility with distinctive sperm pathology. This variant induced alternative splicing by activating a novel splice donor site. The resultant truncated SEPT12 disturbs polymerization of the wild-type SEPT12 in cells. Sperm from cases with this variant were presented with high percentage of abnormal morphology (teratozoospermia) with significant nuclear DNA damage. Our findings indicated a genetic variant of *SEPTIN12* is causally linked to male infertility with distinctive sperm pathology.

### Truncated SEPT12 disrupts SEPT12-related complex in a dose-dependent manner

SEPT12 forms filament- like structure with SEPT4, SEPT6 or SEPT11 *ex vivo* [31], [38], [39]. Previous studies also indicated a mutation in the GTP- binding motif (Gly56) of SEPT12 resulted in large aggregates instated of filamentous structure [32], [39]. However, the components of SEPT12-related complex and how SEPT12 interacts with other SEPTs are not known. In this study, we found truncated SEPT12 disrupts the filamentous structure of wild- type SEPT12 in a dose dependent manner. The ratio of truncated SEPT12 to wild-SEPT12 ranged from 1∶ 1 to 1∶6 (Figure 4), a ratio used to mimic the relative abundance of the alternatively spliced form (1∶1.7). We speculate truncated SEPT12 perturbs self-assembly of wide-type SEPT12 or assembly of wild-type SEPT12 with other SEPTs (e.g. SEPT4, SEPT6 or SEPT11) or other structural proteins during the terminal differentiation of male germline [21], [32], [38], [39], [41]. It is intriguing that wide-type SEPT12 forms filamentous structure wrapping around the nuclear membrane (Figure 4 and 5), a finding consistent with a previous study using the Chinese hamster ovary (CHO) cell, a female germ line cell, co-transfected with SEPT12 and SEPT4 [31]. Moreover, SEPT12 signals were dis-located or disrupted around the sperm nucleus in patients' sperm. It has yet to be tested whether dysfunctional SEPT12 interferes with the integrity of the nuclear membrane.

### Phenotypic variation of SNPs

Recently, Miyakawa et al., suggested *SEPTIN12* as a good candidate gene for male infertility and chose cases with Sertoli- cell-only syndrome (SCOS) [42]. Their study enrolled 140 healthy men and 100 cases with SCOS. They identified eight SNPs (SNP1 to SNP8) in *SEPTIN12*. Among them, three synonymous variants (SNP3 or 210G>A, SNP4 or 225G>A, and SNP6 or 423G>C) were more prevalent in the SCOS patients, but their functional significance still remains to be explored. Two of the 8 SNPs were also found in our study. One is SNP5 or c.332C→A, *pThr111Lys*, but the frequency of c.332C→A did not show significant difference between infertile men and fertile controls in both studies. The other is SNP8 or c.474G→A. The frequency of this SNP did not show any significant difference between patients and controls in the previous study (10.7% vs. 12.0%) [42]. However, in our study, both allelic and genotypic frequencies of c.474A/A were significantly higher in infertile men with abnormal semen parameters (sperm number, motility or morphology). The difference may result from different ethnic backgrounds of enrollees or criteria of patients' selection. It is noteworthy that some fertile men were found to carry c.474A/A, suggesting c.474A/A as a predisposing factor of abnormal spermiogenesis. Indeed, a few infertile men who did not carry c.474G→A (A/A) variant were also presented with similar sperm morphological defects. For these cases, other genetic or environmental factors might be implicated [3], [43], [44], [45], [46], [47]. On the other hand, some fertile controls also carried the c.474G→A (A/A) variant, but the incidence was much lower than the study group. We speculate the c.474G→A (A/A) variant is non-penetrat, a phenomenon common to splicing mutations [48], [49]. Genetic variations of splicing process in human population have been shown to be far more complex than previously observed, and many factors may be accountable for the phenotypic expression of splicing mutations [50], [51], [52]. Incomplete penetrance is common to splicing mutations of many genes [53], [54], [55], [56], [57] and both *cis*- and *trans*-acting modifiers are involved [58], [59], [60]. For SEPT12, the relative abundance of truncated protein may be too low to perturb filament formation in the “non-penetrant” men. Unfortunately the amount of remaining semen samples was not sufficient for us to test this hypothesis.

### SEPT and DNA damage

In our previous study, decreased SEPT12 expression level resulted in significant sperm DNA damage in the mouse [33]. In this study, a SNP of *SEPTIN12* (c.474A/A) was causally linked to the disruption of sperm nuclear integrity and DNA damage. How decreased expression level of SEPT12 and dysfunctional SEPT12 cause nuclear DNA damage still remains to be explored. In yeast, all five septins, Cdc3p, Cdc10p, Cdc11p, Cdc12p, and Shs1p/Sep7p, in the SEPT complex interact with the FHA domain of Rad 53, an important DNA damage checkpoint kinase [61]. Shs1, one of these septins, appears to have an important role in the response to DNA replication stress [61]. In addition, Cdc3p also interacts with BUB2, which is important to maintain a mitotic arrest during kinetochore damage [62]. In mammalian cells, the SEPT 2/6/7 complexes regulate actin organization and are links to the DNA damage checkpoint by accumulation of adaptor protein, NCK, in the nucleus [63]. It deserves to be explored whether the SEPT/SCOS7/NCK pathway is well conserved between different species and different organ systems.

### SEPTIN pathology

Loss of SEPT functions has been implicated in the pathogenesis of many diseases, including neurodegeneration, male infertility and different type of cancers [64]. SEPT1, 2 and 4 are associated with tau-based helical filaments and contribute to the formation of tangles in Alzheimer's disease [65]. Mutations in *SEPTIN9* cause hereditary neuralgic amotrophy in some families [66]. *SEPTIN2*, *5*, *6*, *9* and *11* are involved in reciprocal translocations of myeloid/lymphoid or mixed-lineage leukemia (MLL) gene [67], [68], [69], [70], [71], [72]. *SEPTIN9* was mapped to a region of loss of heterozygosity (LOH) at chromosome 17q25.3 in some cases of sporadic ovarian and breast cancer [73], [74], [75]. Loss of SEPT4 was observed in sperm of patients with asthenozoospermia [12], [13], [28], [29]. In this study, we identified a common *SEPTIN12* variant that may confer susceptibility to defect of spermiogenesis. The characteristic human sperm pathology includes bent tail, abnormal head, immature spermatids, and significant nuclear DNA damage.

## Materials and Methods

### Human samples

The study was approved by the Institutional Review Board of National Cheng Kung University Hospital and Kuo General Hospital. From January 2005 to July 2007, infertile men with abnormal semen parameters were enrolled into the study. They underwent a comprehensive examination, including a detailed medical history, physical examination, hormone profiles and a molecular test for Y-chromosome micro-deletions, as described previously [76]. Patients with Y-chromosomal microdeletion have been excluded from the study. During the same period, we also recruited fertile men with normal semen parameters as control subjects. They were recruited from husbands of women who received regular prenatal care at the University Hospital. All of them had fathered at least 1 child within 2 years without assisted reproductive technologies. All study and control subjects were Han Taiwanese, the major ethnic group in Taiwan (making up more than 95% of the country's population).

### Semen analysis

The ejaculate was obtained by masturbation after a minimum 48 h of sexual abstinence. The assessment of concentration was performed according to the World Health Organization's recommendations using a modified Neubauer chamber and displacement pipettes for proper dilution of the ejaculate [1]. Evaluation of sperm morphology is according to Kruger criteria (normal spermatozoa <14%) and the fourth edition of WHO guidelines for semen analysis [1], [77]. Peroxidase staining was used to detect granulocyte in semen samples and cases with significant leukocytospermia (leukocyte counts >10×10^5^/mL) had been excluded from the study. Abnormal semen parameters included oligozoospermia (sperm count <20×10^6^/mL), asthenozoospermia (percentage of motile sperm <50%), and teratozoospermia (percentage of sperm with normal morphology <14%). The infertile men were recruited if at least one of three major parameters (semen concentration, sperm morphology, and sperm motility) were abnormal.

### PCR and Sequencing

Cases with abnormality in at least one of three major parameters (sperm concentration, motility, and morphology) were subjected to analysis of the *SEPTIN12* gene. Genomic DNA was extracted from peripheral blood samples using a Gentra Puregene Blood Kit (Catalog #158389, QIAGEN, Hilden, Germany.). The entire coding region and exon–intron boundaries of *SEPTIN12* (GenBank accession number NM_144605.3) were analyzed. PCR products were made and visible by using ethidium bromide, followed by sequencing analysis. The oligonucleotide primers are listed in Table S1.

### Cloning, Mutagenesis, Transfection, and RT-PCR

Fragments containing exon 5, intron 5, and exon 6 of human *SEPTIN12* were PCR amplified from human genomic DNAs and cloned into the pEGFP-N1-CMV2 vector. The constructs were confirmed by DNA sequencing. A *SEPTIN12* variant with c.474G→A was prepared using QuickChange Site-directed Mutagenesis Kits (Stratagene, La Jolla, CA) [32]. After transfecting with the plasmids by Lipofectamine 2000 (Invitrogen, Carlsbad, CA), total RNA was extracted from the NTERA-2 d.D1 (NT2D1) cell line, followed by measuring total absorbance at 260 nm for quantification. The RT-PCR conditions and product detection were performed as described in our previous publication [78].

### Separation of the testicular germ cell populations and sperm preparation

Separation of spermatogenic cells was carried out by a centrifugal system based on the density of different types of germ cells, as described previously [79]. After de-capsulation and enzyme digestion of testis biopsies from the case treated with testicular sperm extraction (TESE), germ cell suspensions were filtered through 35 µM nylon filters (Falcon; Becton Dickinson, Franklin Lakes, NJ, USA), followed by centrifugation using a Kubota centrifuge 2010. Germ cells at different developmental stages were collected. Mature spermatozoa were collected from the semen of men with spermatogenic cases and controls. Finally, suspensions were centrifuged with maximal force (2580×g, Kubota 2010) for 10 min, spread on a slide, and air-dried. The slides were then subjected to immuno-fluorescence assay (IFA).

### Immuno-fluorescence assay (IFA)

The protocol of IFA has been described previously [32]. The slide was treated with 0.1% Triton X-100, washed twice with Tris-buffered-saline (TBS), followed by incubation with the anti-SEPT12 antibody (H00124404-B01, Abnova; Taipei, Taiwan) or anti-FLAG antibody (F1804, Sigma, MI, USA) for 60 minutes at room temperature. Following the washing steps with TBS, sections was incubated with goat anti- mouse Alexa Flou 488 or goat anti- mouse Alexa Flou 568 (Invitrogen) for 60 min at room temperature and washed with TBS. Mito-tracker -conjugated with Alexa Fluor 568 (10 mg/ml) (Invitrogen) was used to locate the mid-piece in spermatozoa. 4,6-diamidino-2-phenylindole (DAPI) was used for nuclear staining.

### Motile sperm organelle morphology examination (MSOME) Transmission electron microscopy (TEM) and Atomic force microscopy (AFM)

For bight-field examination, the sperm cells were observed under a high-magnification inverted microscope (Eclipse TE 2000 U; Nikon, Japan) equipped with differential interference contrast microscope (DIC/Nomarski) as described previously [80]. Morphological evaluation was accomplished on a monitor screen and the total calculated magnification was ×8400. Other sperms were air dried on slides after being washed with 1 X phosphate buffered saline (PBS). For TEM studies, spermatozoa were washed in 0.1 M phosphate buffer (pH 7.2), fixed, and further processed according to the protocol described in our previously study [33]. The final sections were counter-stained with lead citrate and uranyl acetate and subjected to observation with a JOEL 1200 TEM (JOEL Institute Inc., Lexington, MA, USA). The AFM examination was performed according to the protocol described previously [40].

### Sperm nuclear DNA damage assays using Acridine orange (AO), Toluidine blue (TB) and Aniline blue (AB) staining

Three assays were used to detect sperm nuclear integrity. The assays included AO, TB and AB staining described in the previous publication [33]. At least 100 spermatozoa were counted for each case per assay.

## Supporting Information

Table S1 **List of human SEPTIN12 primers for sequencing analysis.**
(DOC)
