# Supplementary material for: SEPTIN12 Genetic Variants Confer Susceptibility to Teratozoospermia
Source: PLoS One. 2012 Mar 30;7(3):e34011. doi: 10.1371/journal.pone.0034011 (PMC3316533; doi:10.1371/journal.pone.0034011)
Supplement: Table S1 — List of human SEPTIN12 primers for sequencing analysis. (DOC) [file pone.0034011.s001.doc]

**Supplemental data**

***Table S1. List of human SEPTIN12 primers for sequencing analysis***

| Amplification  Region | Primer Sequence (5’**🡪**3’) | Annealing  Temperature | PCR Product  Size (bp) |
| --- | --- | --- | --- |
| Exon 1-2 | Forward: CAAGTGCAGATGGAGCTCAG  Reverse: TCAGGCCCACCTAAGAGATG | 60℃ | 1038 |
| Exon 3-4 | Forward: CAAGTGCAGATGGAGCTCAG  Reverse: TCAGGCCCACCTAAGAGATG | 60℃ | 366 |
| Exon 5-7 | Forward: CAAGTGCAGATGGAGCTCAG  Reverse: TCAGGCCCACCTAAGAGATG | 58℃ | 701 |
| Exon 8 | Forward: CAAGTGCAGATGGAGCTCAG  Reverse: TCAGGCCCACCTAAGAGATG | 58℃ | 239 |
| Exon 9-10 | Forward: CAAGTGCAGATGGAGCTCAG  Reverse: TCAGGCCCACCTAAGAGATG | 62℃ | 698 |
